# Unveiling a Virulence-Regulating Mechanism in Aeromonas hydrophila: a Quantitative Exoproteomic Analysis of an AraC-Like Protein

**DOI:** 10.3389/fimmu.2023.1191209

**Published:** 2023-05-09

**Authors:** Lishan Zhang, Lina Sun, Ramanathan Srinivasan, Meizhen Lin, Lanqing Gong, Xiangmin Lin

**Affiliations:** ^1^ Fujian Provincial Key Laboratory of Agroecological Processing and Safety Monitoring (School of Life Sciences, Fujian Agriculture and Forestry University), Fuzhou, China; ^2^ Key Laboratory of Crop Ecology and Molecular Physiology (Fujian Agriculture and Forestry University), Fujian Province University, Fuzhou, China; ^3^ State Key Laboratory of Microbial Technology, Shandong University, Qingdao, China; ^4^ Centre for Research, Centre for Materials Engineering and Regenerative Medicine, Bharath Institute of Higher Education and Research, Selaiyur, Chennai, Tamil Nadu, India

**Keywords:** AraC-like transcription factor, quantitative proteomics, *Aeromonas hydrophila*, virulence, ORF02889

## Abstract

Bacterial AraC is a transcription factor family that initiates transcription by recruiting RNA polymerase to the promoter and directly regulating various bacterial phenotypes. It also directly regulates various bacterial phenotypes. However, how this transcription factor regulates bacterial virulence and affects host immunity is still largely unknown. In this study, deleting the orf02889 (AraC-like transcription factor) gene in virulent *Aeromonas hydrophila* LP-2 affected several important phenotypes, such as increasing biofilm formation and siderophore production abilities. Moreover, Δorf02889 also significantly decreased the virulence of *A. hydrophila* and has promising attenuated vaccine potential. To better understand the effects of *orf02889* on biological functions, a data independent acquisition (DIA)-based quantitative proteomics method was performed to compare the differentially expressed proteins between *Δorf02889* and the wild-type strain in extracellular fractions. The following bioinformatics analysis suggested that ORF02889 may regulate various metabolic pathways, such as quorum sensing and ATP binding cassette (ABC) transporter metabolism. Moreover, 10 selected genes from the top 10 decreasing abundances in proteomics data were deleted, and their virulence to zebrafish was evaluated, respectively. The results showed that *ΔcorC*, *Δorf00906*, and *Δorf04042* significantly reduced bacterial virulence. Finally, the following chromatin immunoprecipitation and polymerase chain reaction (ChIP-PCR) assay validated that the promoter of *corC* was directly regulated by ORF02889. Overall, these results provide insight into the biological function of ORF02889 and demonstrate its inherent regulatory mechanism for the virulence of *A. hydrophila*.

## Introduction

AraC is a regulator of the arabinose operon, belonging to the AraC-type transcriptional superfamily of proteins (1). It usually binds to specific promoter sites as monomers or dimers to control the expression of multiple genes and plays a variety of important roles in various bacterial physiological functions, such as carbon metabolism, stress response, and pathogenicity (2, 3). For example, the deletion of the AraC-like transcriptional regulator *yqhc* gene in *Erwinia amylovora* EaUMG3 significantly reduced the expression of several virulence factors (VFs) such as siderophore production and amylovoran production-related genes (4). For another example, the *Staphylococcus aureus* Rbf regulator that belongs to the AraC family negatively regulates the *icaADBC* inhibitor SarR and indirectly inhibits the *ica* operon activator SarX to form RBf-Sarr-Sarx, thus promoting its expression to control the biofilm phenotype of *S. aureus* (5). All available evidence suggests that AraC is crucial in regulating basic biological processes, whereas the instinct regulatory mechanism is still largely unknown.

*Aeromonas hydrophila* is a pathogen that can be transmitted from humans to animals and fish, and is one of the main pathogenic bacteria responsible for bacterial fish diseases. It can cause various illnesses in fish, including gastroenteritis, septicemia, and necrotizing fasciitis (6, 7). The VFs of *A. hydrophila* can be divided into two categories: structural molecules such as lipopolysaccharides (LPS), outer membrane proteins (OMPs), S-layer proteins, and flagella, and virulence-related extracellular secretions, many of which are proteases that are biosynthesized in the cytoplasm and then secreted outside of the cell to infect hosts and cause diseases (8, 9). Previous research has shown that ExsA, an AraC-like protein of the *A. hydrophila* AH3 strain, is a negative regulator of the lateral flagella system and plays an important role in the regulation of the type III secretion system (T3SS) (10). However, few studies have analyzed how the transcription factor AraC regulates the pathogenicity of bacteria in the extracellular fraction level, especially for *A. hydrophila*.

ORF02889 protein is an AraC-like transcriptional regulator in the virulent *A. hydrophila* LP-2 strain that was isolated from diseased silver carp, and its genome was re-sequenced in our library. In this study, the *orf02889* gene was deleted, its rescued strains in *A. hydrophila* LP-2 were constructed, and their physiological functions were evaluated. The results showed that the *Δorf02889* gene significantly affected multiple biological functions, especially for reducing the bacterial virulence in zebrafish. To better understand the regulatory mechanism of ORF02889 on bacterial virulence, a quantitative proteomics method was used to compare extracellular differentially expressed proteins (DEPs) between Δ*orf02889* and wild-type (WT) strains. Subsequent bioinformatics analysis revealed that ORF02889 might regulate various intracellular metabolic pathways. Moreover, the following bacterial experiments also displayed that several selected proteins from proteomics results significantly affected pathogenic virulence. Finally, ChIP-PCR assay showed that several virulence-related genes *corC* were directly regulated by ORF02889. In general, these results provide insight into the biological functions of *orf02889* and demonstrate its regulatory mechanism for *A. hydrophila* virulence.

## Materials and methods

### Bacterial strains and plasmids

In this study, we used the virulent *A. hydrophila* LP-2 strain, which was isolated from a diseased silver carp, and whose genome was re-sequenced in our laboratory. We also used *Escherichia coli* DH5α (GenBank ID: GCA_000982435.1), MC1061 (GenBank ID: LN877770.1), and S17 strains (GenBank ID: GCA_000340255.1), as well as the pRE112 suicide (GenBank ID: JN788332.1) and pBBR1-MCS1 plasmids (GenBank ID: MH238456.1). All of these strains and plasmids were maintained in our laboratory. All strains were cultured using Luria-Bertani (LB) medium (composition: 10 g/L peptone, 10 g/L sodium chloride, and 5 g/L yeast powder). Chrome azurol S (CAS) agar medium was prepared as previously described (11).

### Construction of the gene deletion mutant and rescued strain

The gene deletion strain was constructed based on the principle of homologous recombination as previously described (12). Briefly, the recombinant plasmid containing the target gene’s upper and downstream homologous arms was constructed on the pRE112 suicide plasmid using overlapping PCR technology and introduced into *E. coli* S17. Then, the recombinant suicide plasmid was introduced into the WT strain of *A. hydrophila* through bacterial conjugation. The sucrose lethal gene was used to screen mutant strains. Finally, the deletion strain was verified by PCR and DNA sequencing validation.

The gene-rescued strain was constructed on the broad-host vector pBBR1-MCS1 as previously described (13). After restriction enzyme digestion, the recovery fragment was ligated to the rescue pBBR1-MCS1 plasmid, and then transformed into *E. coli* DH5α competent cells. The single clones were selected from the LB medium plate for bacterial liquid PCR verification, and then the correct recombinant plasmid was extracted and transferred into the previous deletion strain.

### Swimming and swarming ability assays

The swimming and swarming agar plates were prepared using LB medium containing 0.3% and 0.6% agar powder, respectively (14). A single clone of bacterial strains was stabbed and point inoculated on the swimming and swarming agar plates and incubated at 30°C for 6 h and 12 h, respectively. Finally, the swimming and swarming circles’ diameters were measured for three independent repeats.

### Siderophore production assay

Overnight-cultured bacteria were transferred to fresh LB medium at 1% (v/v) and cultured to OD_600_ (optical density at 600 nm) = 1.0 at 200 rpm, 30°C. Then, 5 μl of bacteria solution was spotted onto the CAS agar plate and incubated at 30°C for 16 h. The diameter of the orange halo was observed and measured and was independently repeated three times.

### Detection of biofilm formation by crystal violet staining

Bacterial solutions cultured to OD_600 =_ 1.0 were transferred at 1:20 (v/v) to 96-well microplates with 200 μl in each well. Each sample was technically replicated across four wells per plate and then incubated at 30°C for 24 h (15). After removing the culture medium, 200 μl of 0.1% (w/v) crystal violet solution was added to each well. After 20 min of staining, the solution was removed. Each well was added with 200 μl of 95% ethanol solution and incubated at room temperature for 10 min. The absorbance values under OD 595-nm wavelength were detected by SpectraMax^®^ i3 (Molecular Devices Co., Ltd., Shanghai, China). Each experiment was performed at least three times as biological duplicates.

### The bacterial challenge of mutant strains

The bacterial challenge of mutant strains in zebrafish was performed as previously described (16, 17). Briefly, bacterial strains were cultured overnight and then transferred to fresh LB medium at 1:100 (v/v) for further culture to reach OD_600 =_ 1.0. Then, the bacterial cells from 1 ml of medium were collected by centrifugation, washed with phosphoric acid buffer salt solution (PBS) buffer solution, and then re-suspended in 1 ml of PBS buffer. The bacterial suspension was diluted to a concentration of 1.2 × 10^4^ CFU/ml. Zebrafish fasting for 1 day was randomly divided into 30 fish in each group, and then were intraperitonially injected with the above bacteria concentration at the dose of 10 μl per tail with the injection of the same dose of PBS as the negative control. Challenged fish were monitored for 7 days, and the number of deaths was recorded every day.

### qPCR assay

The quantitative polymerase chain reaction (qPCR) method was used to detect the mRNA expression level of related genes as previously described (18). Briefly, the livers of zebrafish infected with *A. hydrophila* were extracted after intraperitoneal injection of bacterial strains for 3 days. Total RNA from zebrafish liver was extracted by the Trizol-chloroform (TaKaRa Inc., Japan) method. The total RNA was then reverse transcribed into cDNA following the instructions in the Prime Script RT Kit (TransGen Biotech Co., Ltd., Beijing, China). Finally, a real-time PCR detection system (Bio-Rad Inc., CA, USA) was used to detect the expression of related genes by real-time qPCR, and 16S rRNA was used as the internal reference gene. The primers used are shown in **Supplementary Table S1**.

### Extraction of extracellular proteins

Bacterial strains were transferred to 50 ml of LB medium and cultured at 30°C with shaking at 200 rpm until OD_600_ reached 1.0. The supernatant was collected by centrifugation at 10,000 rpm for 10 min at 4°C, and then passed through a 0.22-μm membrane filter. The proteins in the obtained filtrate were precipitated by adding 8% precooled tricarboxylic acid (TCA) (Aladdin Co., Ltd., Shanghai, China) and leaving it overnight at 4°C. After centrifugation at 10,000 rpm for 30 min at 4°C, the proteins were precipitated again from the supernatant by adding 1 ml of precooled acetone and washed three times with acetone. The pellets were dissolved in lysis buffer [6 M urea, 2 M thiourea, and 0.1 M Tris–HCl (pH 8.5) solution with protease inhibitor]. The protein concentration was determined by the Bradford method and stored at −80°C until use.

### Liquid chromatograph mass spectrometer (LC-MS/MS)

Approximately 50 μg of each group of protein samples were taken into an ultrafiltration tube, reduced with 50 mM DTT (dithiothreitol) at 56°C for 40 min. Then, 25 mM IAA (iodoacetamide) was used for alkylation in the dark for 30 min. Then, the protein samples were transferred to an ultrafiltration tube (10 kDa, Millipore, Boston, Massachusetts, USA) and digested by trypsin by using the FASP (filter-aided proteome preparation) method at a ratio of 1:20 (w/v) at 37°C overnight for enzymatic hydrolysis (19). The treated peptide sample was desalted with a C18 column (Waters, Inc., Milford, MA, USA). The desalted polypeptide samples were separated at a flow rate of 60 nl/min by using an EASY-nano-LC chromatography system (Thermo Scientific Inc., Waltham, MA, USA) (20). The separation gradient was as follows: 0–18 min, liquid B (acetonitrile containing 0.1% formic acid) rose linearly from 6% to 12%; 18–77 min, liquid B increased linearly from 12% to 20%; 77–109 min, liquid B rose linearly from 20% to 32%, then rose to 90% within 1 min and remained constant for 120 min. Peptides were analyzed using an Orbitrap Fusion Lumos Tribrid mass spectrometer (Thermo Scientific Inc., Waltham, MA, USA). Specific parameters were set as follows: ion source spray voltage, 2.1 kV; cycle time, 3 s; ion transfer tube temperature, 300°C; scanning range, 300–1,400 m/z; resolution, 120 K; AGC target, 5e5; maximum intensity threshold (IT), 50 ms. The ion trap method was used by MS/MS, the AGC target is 5e3, the maximum IT is 35 ms, and the collision energy is 30%. Data-dependent acquisition (DDA) data from mass spectrum acquisition were imported into Spectronout Pulsar X (biognosys, Schlieren, Switzerland) to establish a DDA database. The database was built using the default optimal parameter “BGS factory setting”. All raw files were searched by Maxquant software (version 1.6.17.0) for database search and data analysis. The mass spectrometry proteomics raw datasets have been deposited to the ProteomeXchange Consortium (http://proteomecentral.proteomexchange.org) *via* the iProX partner repository with the dataset identifier PXD040986.

### Bioinformatics analysis

Based on the mass spectrum data, proteins with a *p*-value less than 0.05 and a difference multiple greater than 2.0 or less than 0.5 were set as differential proteins. GO (Gene Ontology) and KEGG (Kyoto Encyclopedia of Genes and Genomes) enrichment analysis of differential proteins were performed using STRING online software (https://cn.string-db.org/) and visualized by Cytoscape software version 3.9.1 or GOplot and R software. The predicted VFs of DEPs were analyzed with the virulence factors database (VFDB) and visualized with a circular heat map using the TBtool software (21, 22).

### ChIP-PCR assay

The interaction between transcription factor *orf02889* and candidate genes was verified by ChIP-PCR as previously described (23). Briefly, *Δorf02889-C* and *Δorf02889-EV* strains were transferred to 50 ml of LB medium, incubated at 200 rpm overnight, and then centrifuged at 5,000 *g* at 4°C for 10 min. The obtained sample was washed twice with precooled PBS. Formaldehyde solution with a final concentration of 1% was added to the sample for cross-linking for 5 min. Then, glycine with a final concentration of 0.176 M was added to stop the cross-linking. After centrifugation, an appropriate amount of PBS was added for ultrasonic crushing. Then, the supernatant was centrifuged, and the appropriate amount of nickel magnetic beads was added for incubation at 4°C for 2 h. The samples were washed three times with a washing buffer, and then 400 μl of 500 mM imidazole was added to wash and collect. The eluent was added with 40 μl of 10% SDS and 2 μl of 10 mg/ml protease K and incubated at 37°C overnight. The next day, the DNA was extracted using phenol-chloroform. Finally, the purified DNA was verified by PCR using the primers listed in **Supplementary Table S1**.

## Results

### Δ*orf02889* decreases bacterial motility in *A. hydrophila*

**Figure 1:**
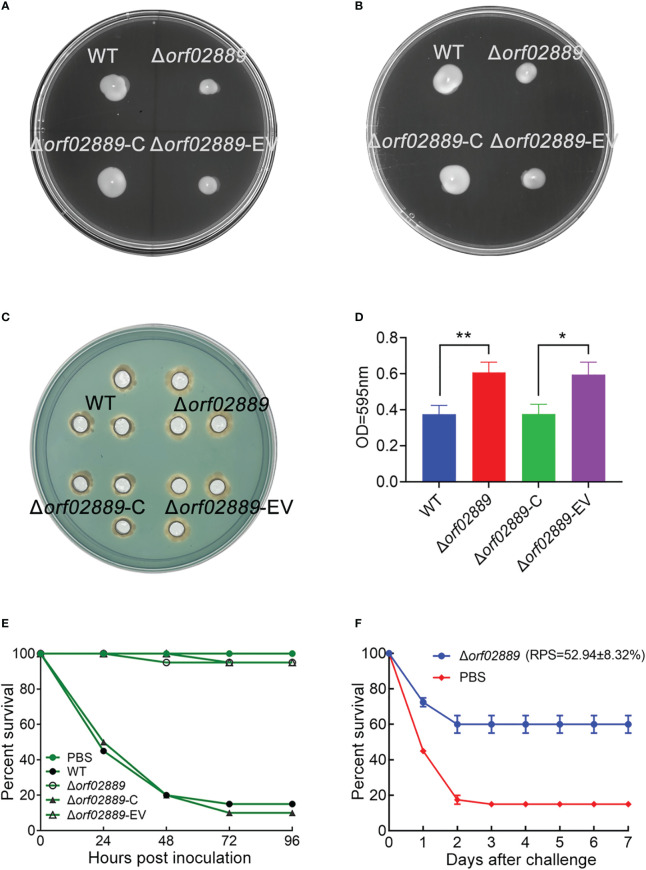
Determination of Δ*orf02889* physiological phenotypes. **(A–D)** The swimming and swarming motility, siderophore production (CAS assay), and biofilm formation of WT and mutant strains. **(E)** Survival rates of zebrafish after challenged with Δ*orf02889*. **(F)** Zebrafish inoculated with Δ*orf02889* and PBS were challenged with *A. hydrophila* LP-2 for 7 days. Data represent the mean ± SEM of *n* ≥ 2 independent replicates. *P < 0.05; **P < 0.01.

To better characterize the physiological phenotypes of ORF02889, the *orf02889* gene was deleted in *A. hydrophila* LP-2, and its rescued strain (Δ*orf02889*-C) with Δ*orf02889* carrying empty vector (Δ*orf02889*-EV) as negative control were constructed in this study (**Supplementary Figure S1**). The swimming and swarming abilities of mutants were assessed on 0.3% and 0.6% semi-solid LB agar plates, respectively, as the locomotion ability of bacteria is closely related to the pathogenicity of pathogenic bacteria. As shown in **Figure 1A**, when compared to the *A. hydrophila* WT strain, the swimming and swarming circles in the Δ*orf02889* strain were significantly reduced. In contrast, these phenotypes were recovered in the rescued strain (**Figure 1B**), indicating that the deletion of the *orf02889* gene affects the locomotion abilities of *A. hydrophila.*

### Δ*orf02889* increases siderophore biosynthesis in *A. hydrophila*

The production of siderophore in *A. hydrophila* was observed using CAS dye solution. As shown in **Figure 1C**, the *Δorf02889* strain produced significantly larger bright orange halos than the LP-2 WT strain, and phenotypic recovery was also observed in rescued strains. This indicates that *orf02889* may negatively regulate the production of siderophore in *A. hydrophila*.

### Effect of Δ*orf02889* on the biofilm formation of *A. hydrophila*

The biofilm formation ability of the *Δorf02889* strain was detected by the crystal violet staining method. As shown in **Figure 1D**, the deletion of the *orf02889* gene significantly increased the biofilm formation ability of *A. hydrophila* when compared to the WT strain. Meanwhile, the biofilm formation ability of the rescued strain was recovered, indicating the important regulatory role of this AraC family regulator on the bacterial biofilm formation in *A. hydrophila*.

### Δ*orf02889* reduces *A. hydrophila* virulence in zebrafish

The effects of Δ*orf02889* on bacterial virulence and immune system-related genes in zebrafish were evaluated in this study. The survival rate of zebrafish on *A. hydrophila* LP-2 infection was 15%, whereas the deletion of *orf02889* caused the survival rate to increase to 95%, indicating that it is a strongly attenuated strain when compared to the results of the PBS injection group as negative control. The behaviors of its rescued strain Δ*orf02889*-C and control strain Δ*orf02889*-EV were similar to those of the WT and Δ*orf02889* strains (**Figure 1E**). Moreover, the overall survival rate of *orf02889* was 60%, and the relative percent survival (RPS) was 52.94 ± 8.32% (**Figure 1F**). It suggested that *orf02889* can be employed as a candidate protein for developing the *A. hydrophila* vaccine.

### Δ*orf02889* stimulates host immunity

The effect of the Δ*orf02889* strain on the zebrafish immune system was also determined. Zebrafish were intraperitoneally injected with WT and Δ*orf02889* mutant strains, respectively, and the transcription levels of genes responsible for pro- and anti-inflammatory cytokines, chemokines, anti-bacterial peptides/enzymes, and antioxidants in zebrafish liver were detected by qPCR after 3 days of injection. As shown in **Table 1**, the deletion of *orf02889* significantly evoked the transcription levels of several pro- and anti-inflammatory cytokines (*il-1β*, *il-6*, and *il-10*), chemokines (*ccl-34α.4*, and *cxcl-8α*), anti-bacterial peptides/enzymes (*defβl-1* and *lyz-c*), and antioxidants (*cat*, *sod-1*, *gstp-1*, and *txndr-1*), downregulated the mRNA levels of *tnf-α* and *tnf-β1*, but did not affect the expression of *cxcl-18b* and *prdx-4* in the host. These results indicate that the *A. hydrophila* Δ*orf02889* attenuated strain could stimulate many host immune-related genes.

**Table 1:** The zebrafish immune response after vaccination with *Δorf02889* and WT strain by qPCR, respectively.

Gene category	Gene name	Δ*orf02889*/WT
Pro- and anti-inflammatory cytokines	*il-1β*	2.69 ± 0.11***
	*tnf-α*	0.23 ± 0.01***
	*tnf-β1*	0.05 ± 0.01***
	*il-6*	1.65 ± 0.10***
	*il-10*	2.31 ± 0.16***
Chemokines	*cxcl-18b*	1.01 ± 0.04
	*ccl-34α.4*	1.96 ± 0.28**
	*cxcl-8α*	1.85 ± 0.19***
Anti-bacterial peptides/enzymes	*defβl-1*	2.01 ± 0.06***
	*lyz-c*	2.14 ± 0.07***
Antioxidant	*cat*	1.85 ± 0.09***
	*sod-1*	1.79 ± 0.07***
	*prdx-4*	1.06 ± 0.07
	*gstp-1*	2.12 ± 0.05***
	*txndr-1*	1.88 ± 0.09**

Values are the mean ± standard deviation of three independent trials. Significant differences between each mutant strain are indicated by asterisks. **p < 0.01**; ***P < 0.001.

### The differential expressions between LP-2 and the Δ*orf02889* strain in extracellular protein samples

**Figure 2:**
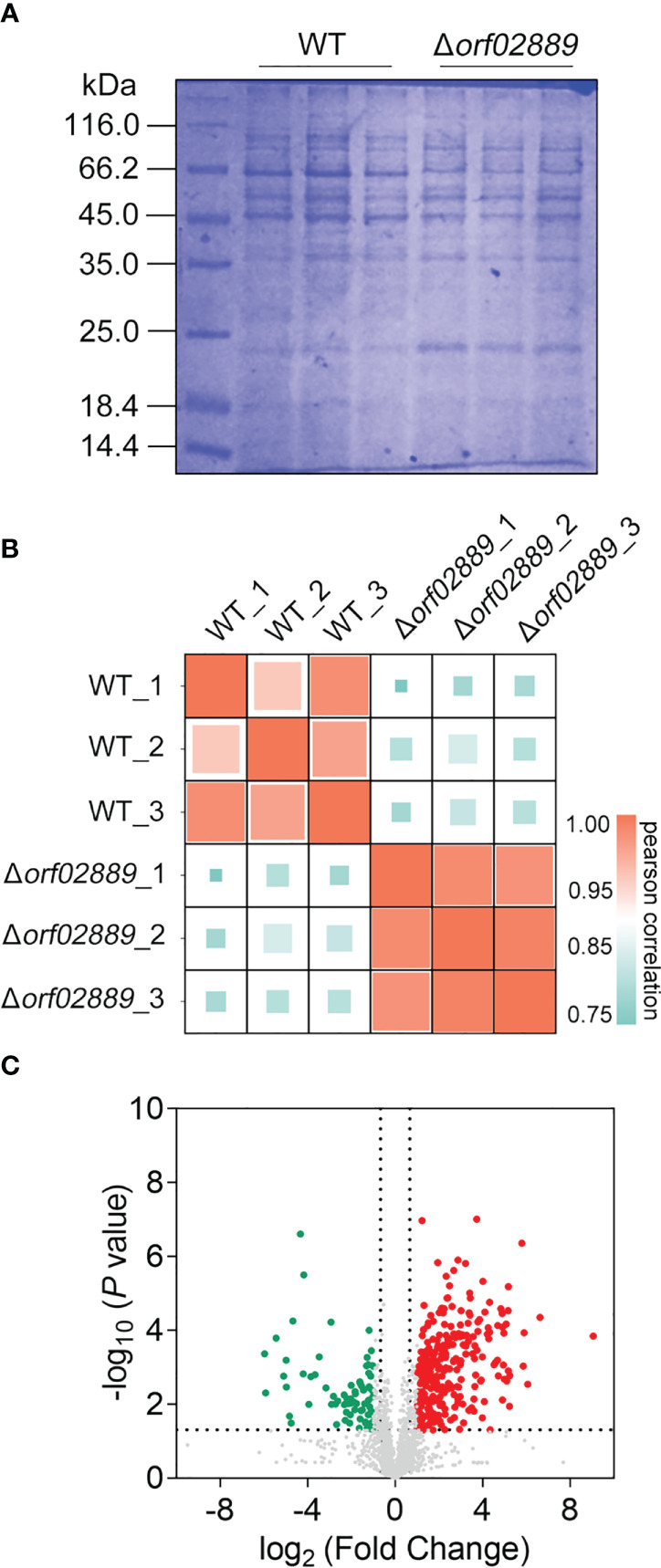
Quantitative proteomic data analysis. **(A)** Coomassie bright blue SDS-PAGE of extracellular proteins extracted from *A*. *hydrophila* LP-2 and Δ*orf02889*; **(B)** Pearson correlation assay of protein intensities for three biological replicates from each group. **(C)** Volcano plot showing the abundance ratios of significant DEPs. Red points represent differentially expressed upregulated proteins, green points represent downregulated proteins, and gray points indicate non-differentially expressed proteins.

Extracellular proteins can directly interact with the host immune system, inducing the body to produce immune protection due to their high immunogenicity. In this study, extracellular protein samples were extracted from *A. hydrophila* LP-2 WT and *Δorf02889* mutant strains, with three biological repeats for each strain (**Figure 2A**). The protein samples in each group were quantitated by LC-MS/MS to compare the protein expression differences between WT and Δ*orf02889*. As shown in **Figure 2B**, correlation analysis for each biological replicate found that all the correlation coefficients *R* were greater than 0.75, indicating that the proteomic data were accurate and reasonable and could be used for further analysis. The further volcano map showed that a total of 2,886 extracellular proteins were identified with 256 DEPs, namely, 112 increasing and 144 decreasing abundance proteins (**Figure 2C**).

### Bioinformatics analysis

**Figure 3:**
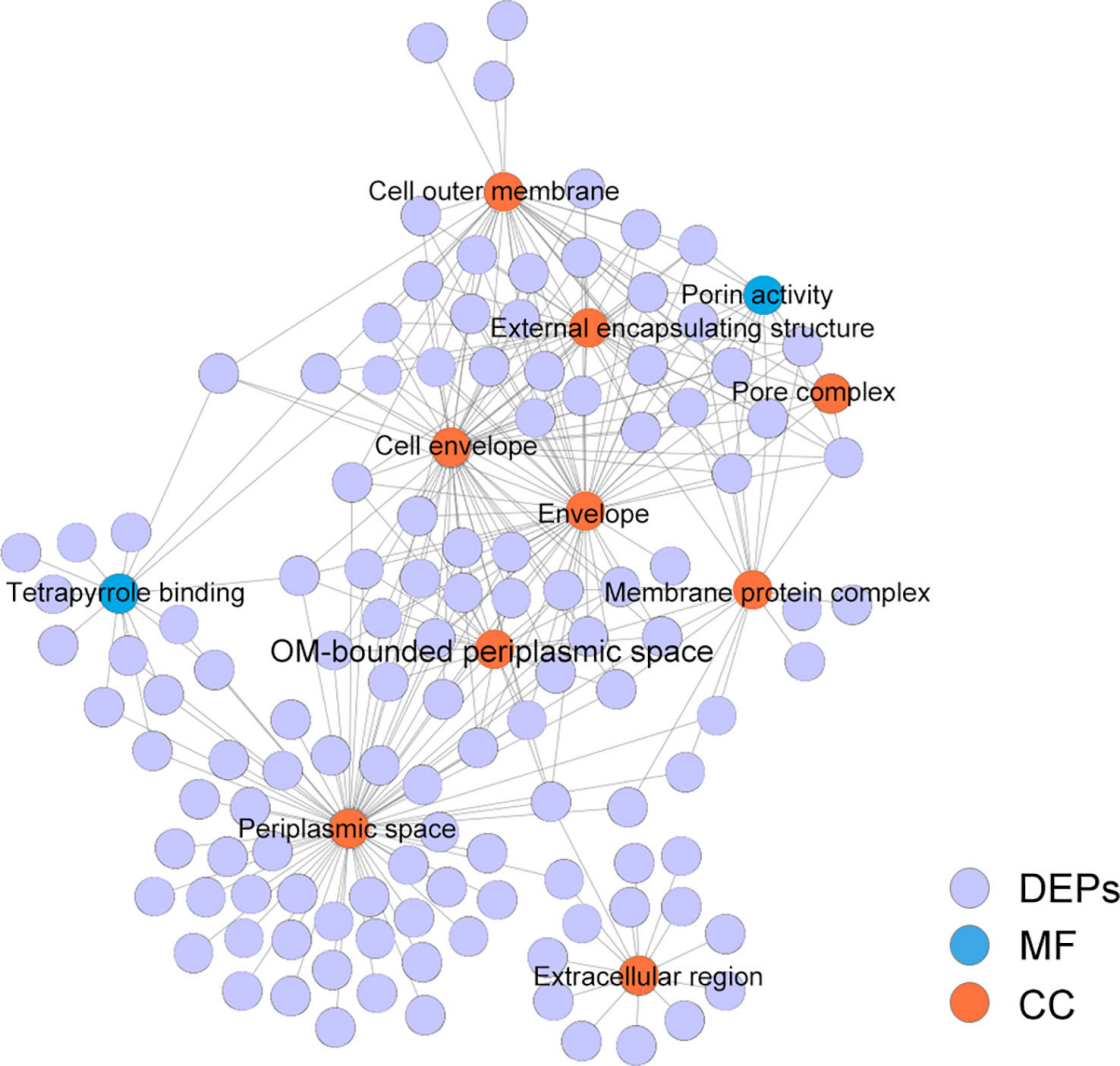
GO enrichment analysis of DEPs between the Δ*orf02889* and WT.

The STRING online software was used for GO enrichment analysis and Cytoscape software for visualization of all the DEPs. The results showed that nine pathways of altered extracellular proteins were enriched in the cell components (CC) category, namely, cell outer membrane, external encapsulating structure, pore complex, cell envelope, envelope, membrane protein complex, OM-bounded periplasmic space, periplasmic space, and extracellular region. In the molecular function (MF) category, two GO terms of altered proteins, namely, porin activity and tetrapyrrole binding, were enriched (**Figure 3**).

### KEGG enrichment and virulence factor analysis

**Figure 4:**
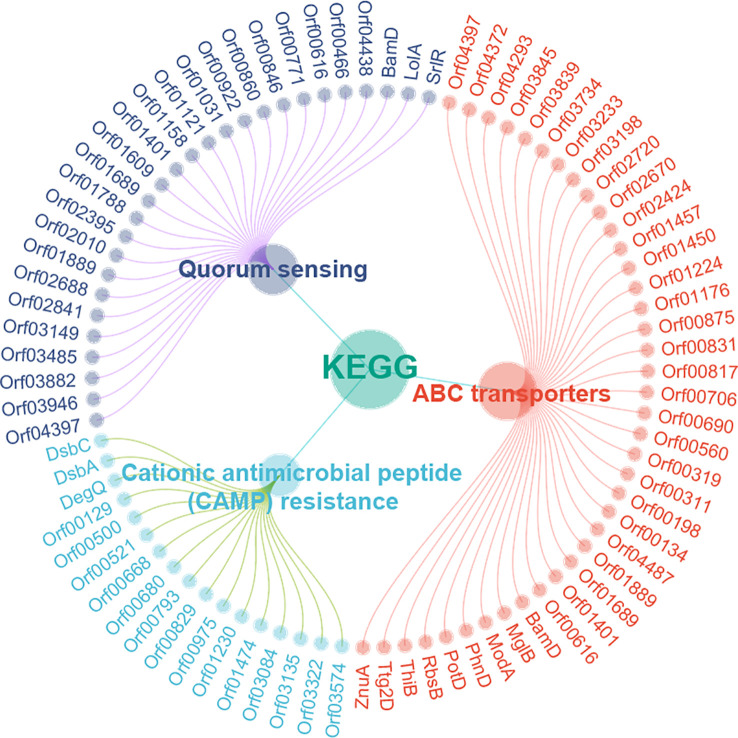
The KEGG pathway enrichment analysis.

The STRING online website was used to enrich and analyze the KEGG metabolic pathways of DEPs. It was found that three KEGG pathways in *A. hydrophila* were enriched after *orf02889* deletion, namely, ABC transporters, cationic antimicrobial peptide (CAMP) resistance, and quorum sensing (**Figure 4**). Among them, at least 39 DEPs were involved in ABC transporters, which is the most enriched KEGG pathway in this study.

**Figure 5:**
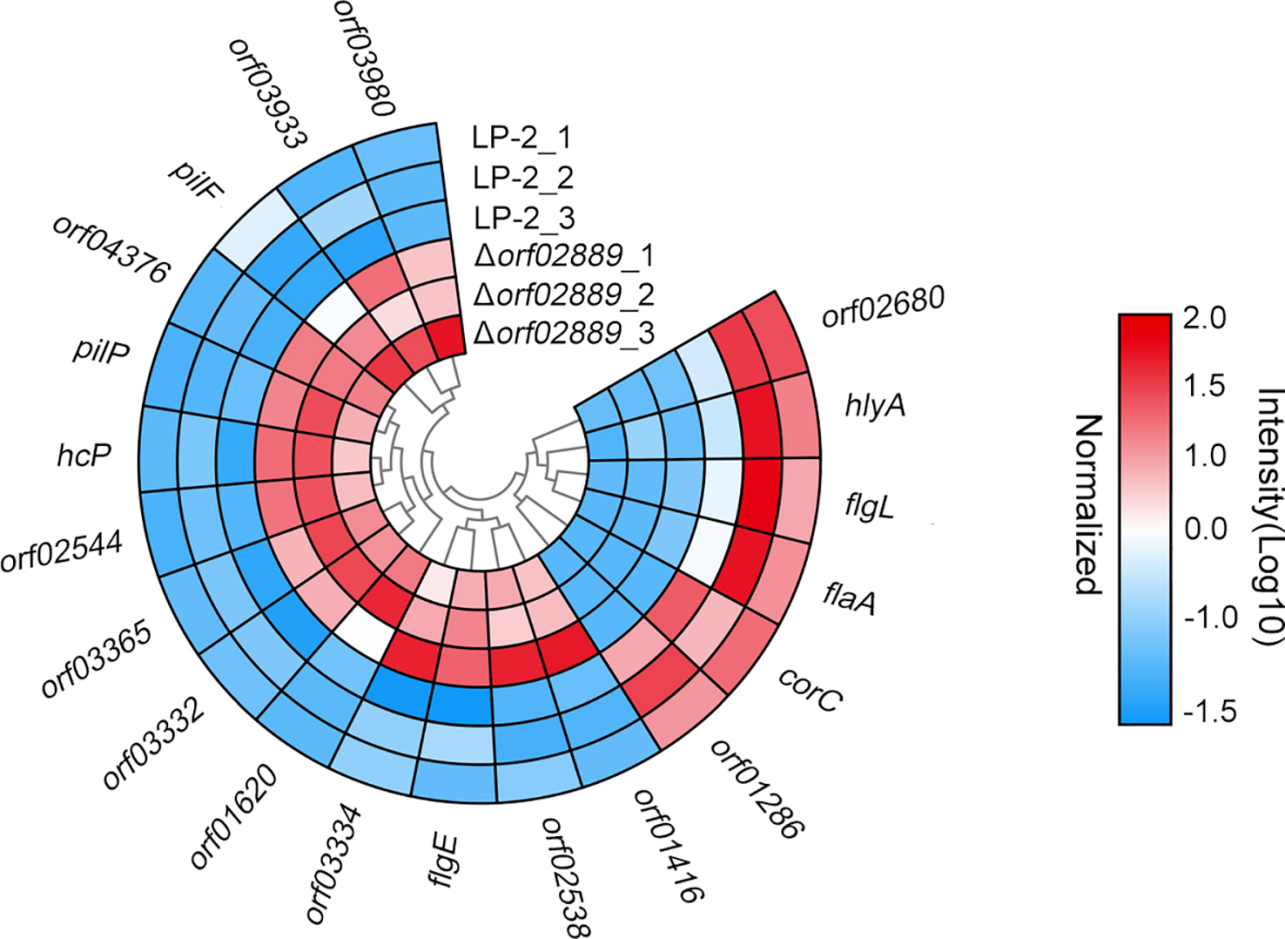
Circular heat map displays the expression of predicted virulence factors in the current proteomics result. The heat map displays the normalized MS intensity of altered proteins in three biological repeats for each group (log10 scale).

The amino acid sequences of DEPs were further submitted to search against the VFDB to identify the well-known VFs in this study. As shown in **Figure 5**, at least 20 VFs were identified, namely, 14 increased proteins and 6 decreased proteins, after *orf02889* was deleted. Interestingly, the protein abundances of these VFs were fluctuant, indicating that the virulence behavior should be a comprehensive complicated regulatory mechanism.

### The effects of selected genes regulated by ORF02889 on bacterial virulence

**Figure 6:**
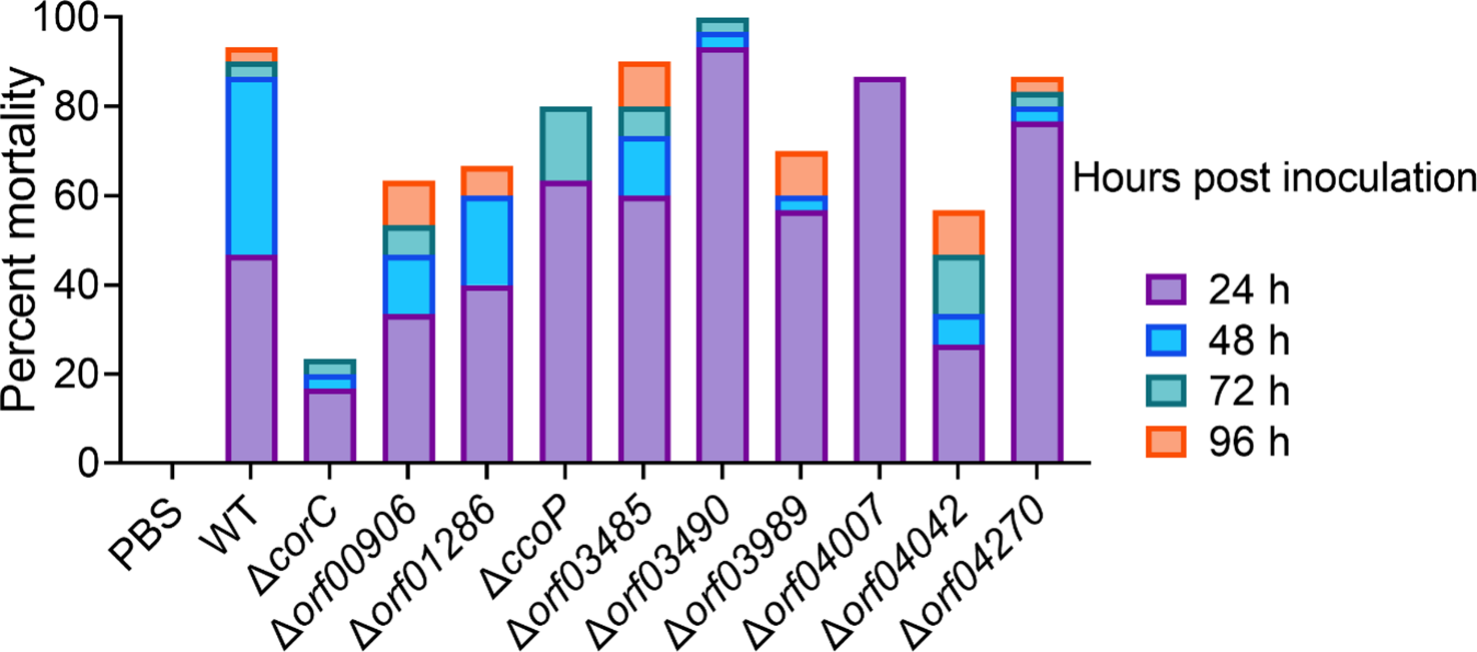
Cumulative zebrafish mortality in 96 h after a challenge by associated gene deletion strains.

To better understand the role of the transcription regulator *orf02889* in the virulence of *A. hydrophila* LP-2, we first selected 10 proteins [ORF00560(CorC), ORF00906, ORF01286, ORF02228(CcoP), ORF03485, ORF03490, ORF03989, ORF04007, ORF04042, and ORF04270] with the highest downregulated abundances from the proteomic data for gene knockout and further functional verification. These protein-coded genes were successfully knocked out in *A. hydrophila* LP-2 (**Supplementary Figure S2**), and their effects on the virulence of *A. hydrophila* were evaluated by intraperitoneal injection of mutants into zebrafish. The results showed that the cumulative mortality rate of Δ*corC*, Δ*orf04042*, and Δ*orf00906* fell by 70%, 36.7%, and 30% when compared to *A. hydrophila* LP-2 with a mortality rate of 93.33%, respectively, indicating a significant reduction in virulence of these mutants. Besides these, the mortality rate of the remaining gene deletion strains was similar to or a little lower than that of *A. hydrophila* LP-2 (**Figure 6**). These results suggest that CorC, ORF00906, and ORF04042 may be novel *A. hydrophila* virulence effectors and may play an important role during bacterial invasions.

### *orf02889* directly regulates the transcription of *corC*

**Figure 7:**
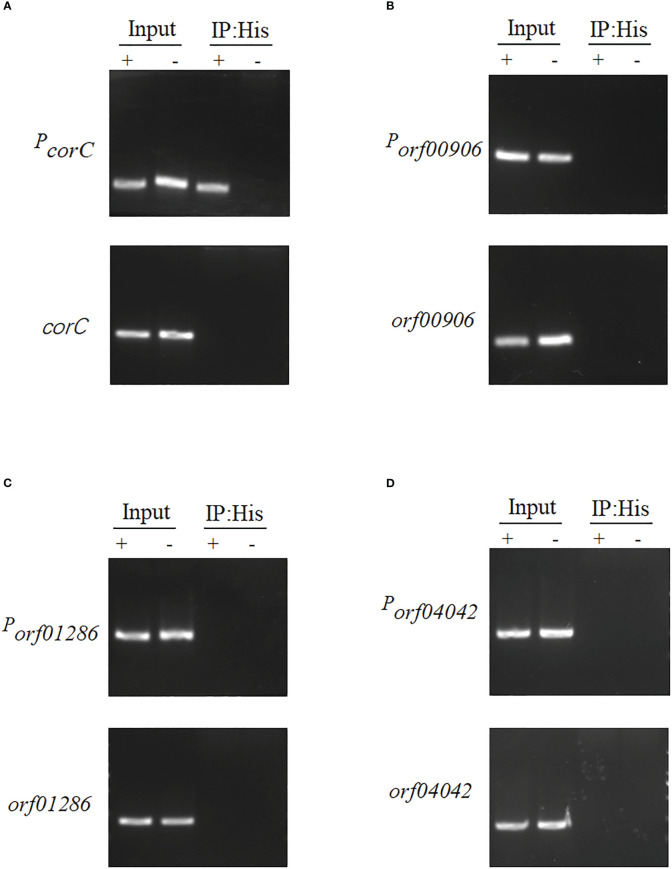
The binding assay of *orf02889* with the promoters of four candidate genes. **(A–E)** ChIP-PCR analysis of the binding of *orf02889* and the promoter fragments of *corC*, *orf0906, orf01286*, and *orf04042*. The *A. hydrophila* chromatin was immunoprecipitated without or with anti-His antibody (Input or IP: His) for PCR with the target primer pairs, respectively. The promoter fragments of candidate genes are marked as P_gene name_; the *orf02889* complementary strain was marked as “+”; Δ*orf02889* carrying empty vector was marked as “-”.

The ChIP-PCR method was used to detect the binding ability of four candidate genes with *orf02889*; the virulence of these related mutants was attenuated in zebrafish infection. They are magnesium and cobalt efflux protein CorC (ORF 00560), acetyl-coenzyme A synthetase (ORF00906), diguanylate cyclase/phosphodiesterase (ORF01286), and ferrichrome-iron receptor (ORF04042). As shown in **Figure 7**, all candidate genes (*corC, orf00906, orf01286*, and *orf4042*) and their related predicted promoter regions (*P_corC_, P_orf00906_, P_orf01286_*, and *P_orf4042_*) can be detected in the Δ*orf02889*-C input sample (positive control). All immunoprecipitated (IP) DNA fragments of candidate genes cannot be amplified in the Δ*orf02889-C* sample as expected using the designed primer pair for target gene amplification. Meanwhile, *P_orf00906_, P_orf01286_*, and *P_orf4042_* were not amplified, but *P_corC_* can be detected in IP samples. These results indicate that *orf02889* may affect the *A. hydrophila* virulence by directly regulating the expression of the *corC* gene.

## Discussion

Numerous studies have demonstrated that AraC-like transcription factors play a crucial role in regulating various physiological functions of bacteria. They are mainly involved in the metabolism of carbon sources, responses to environmental stress, and pathogenesis. For example, the *S. aureus* Rsp protein, which belongs to the AraC/XylS transcription regulatory factor (AFTRs) family, can promote its own expression by directly binding to the promoter regions of *hla* and *psm* (pore toxin) to positively regulate *S. aureus* virulence determinants, indicating its important role in the regulation of bacterial virulence (24).

ORF02889 is a member of the bacterial transcription factor AraC family in virulent *A. hydrophila* LP-2. Our current physiological phenotype assays of *orf02889* mutants showed that this transcription factor played important roles in various bacterial biological functions. For example, it reduced motility ability while increasing biofilm formation and siderophore biosynthesis capabilities. Subsequent quantitative proteomics studies have shown that *Δorf02889* strains affect the ABC transport system. Reduced motility in bacteria can increase the interaction time between the bacterium and its substratum, thereby promoting biofilm formation (25). However, ferriferous carriers are necessary not only for surface transport, but also for bacterial biofilm formation. In addition, Carol et al. found that the surface motility of bacteria is prevented under high-iron conditions, but biofilm formation is promoted by inducing the formation of more structured and thicker biofilms than those formed under low iron levels. It has also been shown that *Sinorhizobium meliloti* has control mechanisms that inversely regulate swarming and biofilm formation, and facilitate bacterial invasion of the host (26).

Moreover, the deletion of *orf02889* significantly decreased the virulence of *A. hydrophila* LP-2 in zebrafish, and it may be a promising attenuated vaccine against *A. hydrophila* infection. This is because the injection of the mutant strain can provoke the host immune response, and the RPS of the immunized zebrafish was higher than 50% when challenged with the *A. hydrophila* LP-2 strain.

To better understand how ORF02889 regulates bacterial virulence, we first used quantitative MS to characterize the effect of *Δorf02889* on the exoproteome of *A. hydrophila* cells. The results showed that 380 extracellular proteins were differentially expressed in *Δorf02889* strains, among which 295 proteins were upregulated and 85 were downregulated. Subsequent bioinformatics analysis indicated that *orf02889* might be involved in the regulation of the quorum sensing and ABC transporters’ mechanisms. Previous research has reported that AraC family transcription factors such as *Pseudomonas aeruginosa* VqsM and *Yersinia pestis* YbtA directly regulate quorum sensing and ABC transport systems, which is consistent with our current results (27, 28).

In this study, at least 20 altered proteins are predicted as VFs based on the homology comparison in the VFDB. For example, *Vibrio cholerae* hemolysin HlyA can influence the invasion ability of the bacteria by regulating the hemolytic activity of the bacteria, thus participating in the pathogenic process of the bacteria (29). In this study, the abundance of HlyA was decreased in *Δorf02889* strains, which indicates that ORF02889 may affect *A. hydrophila* virulence by regulating *hlyA*. However, of these altered proteins, only six predicted VFs were downregulated in *Δorf02889* strains, suggesting that these proteins may be more important in contributing to ORF02889-mediated *A. hydrophila* virulence. To further investigate the virulence regulatory mechanism of this AraC-like transcription factor, we selected 10 altered proteins that have top decreasing abundances in our exoproteomic data for further functional validation, including the above two predicted VFs, ORF01286 and CorC. ORF01286 is a diguanylate cyclase/phosphodiesterase containing a GGDEF domain that synthesizes bacterial second messenger bis-(3′–5′)-cyclic dimeric guanosine monophosphate (c-di-GMP) controls and plays an important role in the regulation of bacterial flagella movement and virulence (30, 31); CorC is a magnesium and cobalt efflux protein that plays an important role in Mg^2+^ and Co^2+^ homeostasis in various bacterial species (32). Although previous research documented that CorA but not CorC in *Salmonella enterica* serovar Typhimurium is important for the invasion of Caco-2 epithelial cells, the deletion mutation of *Francisella tularensis* FTL_0883, a homolog of CorC, attenuated *F. tularensis* subsp. *holarctica* live vaccine strain (LVS) replication within macrophages *in vitro*, suggesting that the role of CorC on virulence has species specificity (33, 34).

Moreover, we deleted the selected 10 genes, respectively, and evaluated their effects on bacterial virulence. Our results displayed that at least three mutants (*ΔcorC*, *Δorf00906*, and *Δorf04042*) significantly reduced bacterial virulence during the zebrafish infection when compared with the WT strain, especially for *ΔcorC*, which increased the zebrafish survival rate to 76.67%. The following ChIP-PCR analysis further confirmed that *orf02889* can directly bind to the promoter of *corC*, but *orf00906*, *orf01286*, and *orf04042* did not. Therefore, our results indicate that the AraC-like transcription factor *orf02889* may regulate the expression of several VFs, such as CorC, and play an important role in *A. hydrophila* virulence.

## Data availability statement

The datasets presented in this study can be found in online repositories. The names of the repository/repositories and accession number(s) can be found at http://www.proteomexchange.org/,PXD040986.

## Ethics statement

The animal study was reviewed and approved by the Fujian Agriculture and Forestry University Animal Care and Use Committee (Certification Number: CNFJAC0027).

## Author contributions

XL conceived and supervised the project. LZ constructed strains, performed the experimental work, and drafted the manuscript. LS and ML contributed to quantitative proteomics and data analysis. RS and LG improved the manuscript. All authors contributed to the article and approved the submitted version.
