# Supplementary material for: Unveiling a Virulence-Regulating Mechanism in Aeromonas hydrophila: a Quantitative Exoproteomic Analysis of an AraC-Like Protein
Source: Front Immunol. 2023 May 9;14:1191209. doi: 10.3389/fimmu.2023.1191209 (PMC10203433; doi:10.3389/fimmu.2023.1191209)
Supplement: Supplementary file 1 [file DataSheet_1.docx]

**Figure S1**


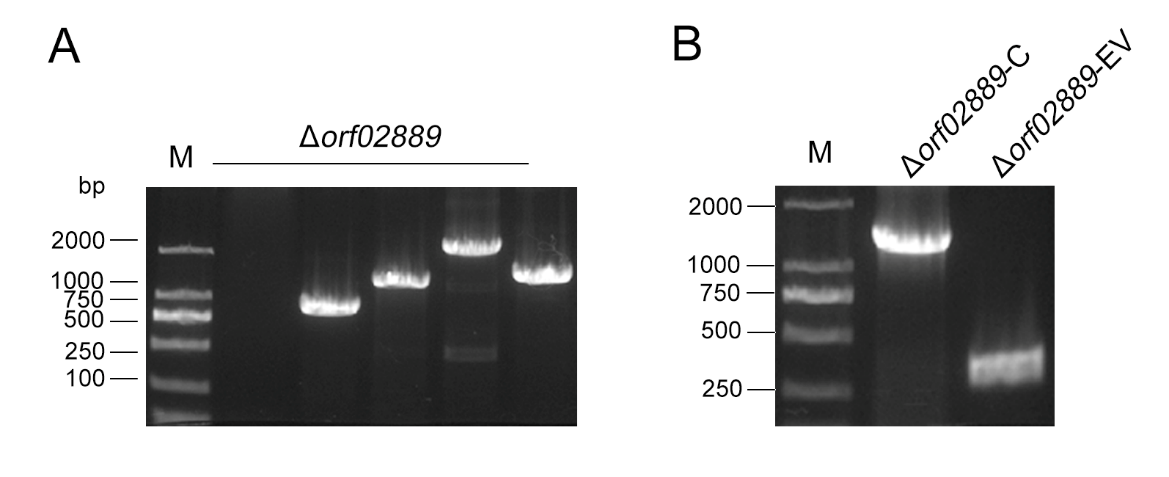


**Figure S1. Construction of *orf02889* deletion and rescue strains.** (A) M: DL2000 DNA Marker; The lanes from left to right were deletion strain P5P6, P5P6 positive control, deletion strain P7P8, P7P8 positive control and deletion strain verification. Here, the LP-2 genomic DNA was used as a template for the positive control. P5/P6 were the upstream and downstream primers of the target gene), P7/P8 were primers on both sides of the upstream and downstream homologous arms, and the primers of the validation common-F/R were derived from the upstream and downstream of the last sequence fragment of the LP-2 genome, with the size of about 1400bp, which were used to verify whether the deletion strain was LP-2. (B) M: DL2000 DNA Marker; The lanes from left to right were F/R rescued strains and F/R empty vector strains.

**Figure S2**


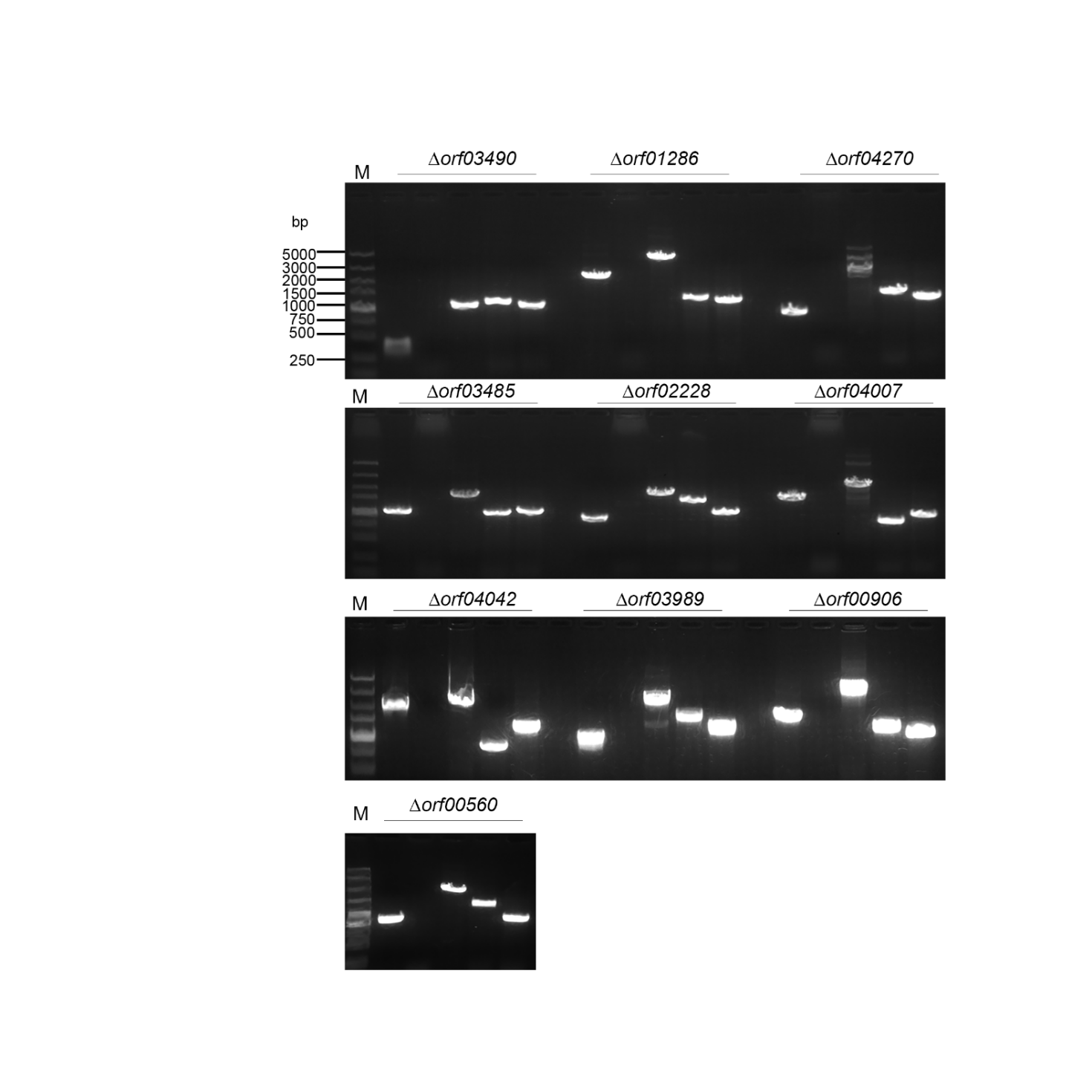


**Figure S2. Construction of related gene deletion strains.** M: DL2000 DNA Marker; Three pairs of primers were used for colony PCR validation of each deletion strain, namely P5/P6 (upstream and downstream primers of the target gene), P7/P8 (located on both sides of the upstream and downstream homologous arms, respectively) and validation primers common-F/R (primers derived from the upstream and downstream of any sequence fragment of the LP-2 genome, with a size of about 1000bp.To verify whether the deletion strain is LP-2). During agarose gel detection, each strain of deletion strain had 5 lanes, which were P5P6 positive control, deletion bacteria P5P6, P7P8 positive control, deletion strain P7P8 and deletion strain verification from left to right. Here, the LP-2 genomic DNA was used as a template for the positive control.
