# Supplementary material for: Unveiling a Virulence-Regulating Mechanism in Aeromonas hydrophila: a Quantitative Exoproteomic Analysis of an AraC-Like Protein
Source: Front Immunol. 2023 May 9;14:1191209. doi: 10.3389/fimmu.2023.1191209 (PMC10203433; doi:10.3389/fimmu.2023.1191209)
Supplement: Supplementary file 2 [file Table_1.docx]

**Supplementary Table S1. The primer pairs used in this study.**

| Gene | Oligonucleotide sequence 5′-3′ | Purpose |
| --- | --- | --- |
| *orf02889*-P1  *orf02889*-P2  *orf02889*-P3  *orf02889*-P4  *orf02889*-P5  *orf02889*-P6  *orf02889*-P7  *orf02889*-P8  *orf02889*-F  *orf02889*-R  *il-1β*-F  *il-1β*-R  *tnf-α*-F  *tnf-α*-R  *tnf-β1*-F  *tnf-β1*-R  *il-6*-F  *il-6*-R  *il-10*-F  *il-10*-R  *cxcl-18b*-F  *cxcl-18b*-R  *ccl-34α.4*-F  *ccl-34α.4*-R  *cxcl-8α*-F  *cxcl-8α*-R  *defβl-1*-F  *defβl-1*-R  *lyz-c*-F  *lyz-c*-R  *cat*-F  *cat*-R  *sod-1*-F  *sod-1*-R  *prdx-4*-F  *prdx-4*-R  *gstp-1*-F  *gstp-1*-R  *txndr-1*-F  *txndr-1*-R  *orf03490*-P1  *orf03490*-P2  *orf03490*-P3  *orf03490*-P4  *orf03490*-P5  *orf03490*-P6  *orf03490*-P7  *orf03490*-P8  *orf01286*-P1  *orf01286*-P2  *orf01286*-P3  *orf01286*-P4  *orf01286*-P5  *orf01286*-P6  *orf01286*-P7  *orf01286*-P8  *orf04270*-P1  *orf04270*-P2  *orf04270*-P3  *orf04270*-P4  *orf04270*-P5  *orf04270*-P6  *orf04270*-P7  *orf04270*-P8  *orf03485*-P1  *orf03485*-P2  *orf03485*-P3  *orf03485*-P4  *orf03485*-P5  *orf03485*-P6  *orf03485*-P7  *orf03485*-P8  *orf02228*-P1  *orf02228*-P2  *orf02228*-P3  *orf02228*-P4  *orf02228*-P5  *orf02228*-P6  *orf02228*-P7  *orf02228*-P8  *orf00560*-P1  *orf00560*-P2  *orf00560*-P3  *orf00560*-P4  *orf00560*-P5  *orf00560*-P6  *orf00560*-P7  *orf00560*-P8  *orf04007*-P1  *orf04007*-P2  *orf04007*-P3  *orf04007*-P4  *orf04007*-P5  *orf04007*-P6  *orf04007*-P7  *orf04007*-P8  *orf04042*-P1  *orf04042*-P2  *orf04042*-P3  *orf04042*-P4  *orf04042*-P5  *orf04042*-P6  *orf04042*-P7  *orf04042*-P8  *orf03989*-P1  *orf03989*-P2  *orf03989*-P3  *orf03989*-P4  *orf03989*-P5  *orf03989*-P6  *orf03989*-P7  *orf03989*-P8  *orf00906*-P1  *orf00906*-P2  *orf00906*-P3  *orf00906*-P4  *orf00906*-P5  *orf00906*-P6  *orf00906*-P7  *orf00906*-P8  P_corC_-F  P_corC_-R  P*_orf00906_*-F  P*_orf00906_*-R  P*_orf01286_*-F  P*_orf01286_*-R  P*_orf04042_*-F  P*_orf04042_*-R | CGATCCCAAGCTTCTTCTAGACAGGTCGAGCGAACGATAGC  CGACCACTGATATGCGTCCTCCTGCAGGTG  AGGACGCATATCAGTGGTCGCCTCCTGATGA  ATGAATTCCCGGGAGAGCTCTCACAATTGTGATCCATCCGG  CATATGCAGTGGGAAGAAGTG  GGTGGACTTGCTGTTGAAAC  TGATAGGCCAGCAACTGGGT  ACCATCGCCAACTACCAGTAC  GTCGACGGTATCGATAAGCTTGATCTTGGTTCCTTCTGTGGTCCT  CGCTCTAGAACTAGTGGATCCTCAATGGTGATGGTGATGATGGGAGGCGACCACTGCTTGCGCCC  TCAAACCCCAATCCACAGAG  TCACTTCACGCTCTTGGATG  AGAAGGAGAGTTGCCTTTACCGCT  AACACCCTCCATACACCCGACTTT  GTGGACACTCGATCGCTACC  CCAGCTTTGGGGTGAAGTCT  TCAACTTCTCCAGCGTGATG  TCTTTCCCTCTTTTCCTCCTG  CCCTATGGATGTCACGTCATG  CATATCCCGCTTGAGTTCCTG  CTGCTGCTCGCGGTAGTTTA  TCAACTTTGTCGCAGTTTGG  TGCAGCTCAACCAGAAGATG  CTTTGACGCATGGAGGATTT  CTTCCCTCCAAGCCCACAC  CTTCCCTCCAAGCCCACAC  CTTCCCTCCAAGCCCACAC  TTTGCCACAGCCTAATGGTCCGAA  AAGCAGGTTTAAGACCCACCGAGT  AAGTCTGAACAGGCCACTTTGCAC  CCAAGGTCTGGTCCCATAAAG  GCTCAACCTCCGCGAAATA  AGGTGACTGGTGAAATTACTGG  GTCTCACACTATCGGTTGGC  AGAGGGAGCAGGAGTGTTATA  GGTGCTGGTTTTGAGATTTTGG  TTGAAGAGTGGATGAAGGGC  TTGAAGAGTGGATGAAGGGC  CAAACAAGCGTGGCAAAGAG  ACAGTAGGGCAAAGAGAACAG  CATGAATTCCCGGGAGAGCTCATCGCTGTCCTCCTGACCG  TGGCTGGCACATCATATCAGGGTTTTTACATGCATC  CTGATATGATGTGCCAGCCATCAAGGTGAT  CGATCCCAAGCTTCTTCTAGAACCTTGACGAACTCTTCGGACA  TGATGCTGCCTGGAGGTAAC  TTGATGGCTGGCACTCACTC  CAGCTCGTCGAGTATCACCT  GGTCGTGGTCATGACCATGA  CATGAATTCCCGGGAGAGCTCTCATCGGGATGGAAGAGTCG  ACACGTTCACGCTGAACCAAGCATGGG  TTGGTTCAGCGTGAACGTGTGAAACGAGAAGGG  CGATCCCAAGCTTCTTCTAGAATGATCCTCATCTACCTGCTGGTG  CCATGCTTGGTTCAGCGATG  TCAGCGTCCTTTCATCAGCG  GATCCATCTGACGGAAGATG  TGATCCTCATCTACCTGCTG  CATGAATTCCCGGGAGAGCTCTACTTTATCGGCGGCAGCC  CGACCCGGTATTCATCTGTTACTCCTTCACAGTGATAGCG  AACAGATGAATACCGGGTCGGCATG  CGATCCCAAGCTTCTTCTAGAAGCACCACCACCTTGGCG  AGGAGTAACAGATGCGACTG  GATTTCCTTGGGTACGCCAA  GAACCACATGCTGGTGATGC  GATGCACGCCTCTATGTTGG  CATGAATTCCCGGGAGAGCTCTGCAATACCAGGTCGAGAAGATG  GTGACAAGCCATCCTTGCAATTGATTTT  TTGCAAGGATGGCTTGTCACCCTCCCCGGGAT  CGATCCCAAGCTTCTTCTAGAAGTACCGATCAAAAGGTGCGC  GTGGCTGAAAGCAAACCTCG  CTTGCGATAGATGGTGGAAGG  AGATCAACGAGCTGACCAAG  AGGAGCTTTATCTCGCCATC  CATGAATTCCCGGGAGAGCTCAGCGTACCGGTCCGGATC  TGTATTGTTTCGCTCCTGCGTTC  CGCAGGAGCGAAACAATACATGTGATAAACACGTGATTACTCAGC  CGATCCCAAGCTTCTTCTAGATGTCGAGCAGCAGGCGAT  GATAACAAGAACGCAGGAGC  GGGACAAGCTGTAAACGTAGG  GACATCTTCATCCGCGAAGG  ATGACTCAGCGTCCTTTGGG  CATGAATTCCCGGGAGAGCTCAGTTCATCGTGAGCATCCGC  ATGCATCGCTGGCAGTGTAATGAATTT  TTACACTGCCAGCGATGCATCAGGATCTGGTGACGC  CGATCCCAAGCTTCTTCTAGAACTGATACTCGGACGCCAGC  GGAGTGCTGTTGCAGTATGG  CAAACTCCGCAGGTTAGCTG  AGACCATCTGGAAATCGGTC  TGGCCCTGTGTATTTGTCTC  CATGAATTCCCGGGAGAGCTCTATCGATGTGGAATATTTGCAAGG  CTGGCTCAGTACTCTTTCCTTATCTCTTTGTTTATTTTG  AGGAAAGAGTACTGAGCCAGGTAACGTTTTCAG  CGATCCCAAGCTTCTTCTAGAAGCTTGTCGTCACCGCCTT  ATGGCTTGTCATATGCGCAC  AAAACGTTACCTGGCTCAGTC  AGTCTTCATCAATCGCCACCAC  AGATCCACGTTGAAGAAGTCGC  CATGAATTCCCGGGAGAGCTCGACAGCTACCTGCTCTCCATCG  GGTGCTTCATGTTGCGTGTTGACTCTTGAAGAAG  AACACGCAACATGAAGCACCGAGCGATGAA  CGATCCCAAGCTTCTTCTAGAACCAGTTGCCACGGCAGG  TTCAGACCATCAGGTCGCTT  TTCATCGCTCGGTGCTTCAT  TTCCTGTCCGACGACAAGAC  CGTGCAGCTTGTTCCACATC  CATGAATTCCCGGGAGAGCTCATTGTGATTAATGCAGTTGGCG  CCTTGCTCATATGCCAGTACCGACCAGCAT  GTACTGGCATATGAGCAAGGCATGGCTGAT  CGATCCCAAGCTTCTTCTAGAC  TGGCTATTGGTGCTGCTGTG  GCCATGCCTTGCTCATAACC  ATGTAGGTCTGATGGTGACG  TTATTGCTGTGACGACTCGC  CATGAATTCCCGGGAGAGCTCTGCAGGTGACGCTCATCAGTT  CGCATGAGTGTCACTCCTTTGAATACATTCTTGT  AAAGGAGTGACACTCATGCGCACCATATTTCCG  CGATCCCAAGCTTCTTCTAGAATCTCATCGAGCGTCTTGCTG  TGAGCGAGAGCAAGGTCTAC  GCATGATCAAGCCGCTTCTG  GCCAGTTTCTGCAGTCACTG  GAAATCCACCTGACCCATGG  GGATCTTCCAGAGATGCCGGATTTGACTTGGTAGAAG  CTGCCGTTCGACGATACTGCAACAGCACTCCGAGTAGA  GGATCTTCCAGAGATGTCGCTAAAACGACGTTTTACCC  CTGCCGTTCGACGATCTTTGAATACATTCTTGTAATGATTAGGTAA  GGATCTTCCAGAGATCTGTTGCGCATCTCCCATTC  CTGCCGTTCGACGATGCTGAACCAAGCATGGGCA  GGATCTTCCAGAGATCTTGCTGACAGGGCGCCC  CTGCCGTTCGACGATGTTGCGTGTTGACTCTTGAAGAAG | Gene deletion  Gene deletion  Gene deletion  Gene deletion  Gene deletion  Gene deletion  Gene deletion  Gene deletion  Complementary  Complementary  qPCR  qPCR  qPCR  qPCR  qPCR  qPCR  qPCR  qPCR  qPCR  qPCR  qPCR  qPCR  qPCR  qPCR  qPCR  qPCR  qPCR  qPCR  qPCR  qPCR  qPCR  qPCR  qPCR  qPCR  qPCR  qPCR  qPCR  qPCR  qPCR  qPCR  Gene deletion  Gene deletion  Gene deletion  Gene deletion  Gene deletion  Gene deletion  Gene deletion  Gene deletion  Gene deletion  Gene deletion  Gene deletion  Gene deletion  Gene deletion  Gene deletion  Gene deletion  Gene deletion  Gene deletion  Gene deletion  Gene deletion  Gene deletion  Gene deletion  Gene deletion  Gene deletion  Gene deletion  Gene deletion  Gene deletion  Gene deletion  Gene deletion  Gene deletion  Gene deletion  Gene deletion  Gene deletion  Gene deletion  Gene deletion  Gene deletion  Gene deletion  Gene deletion  Gene deletion  Gene deletion  Gene deletion  Gene deletion  Gene deletion  Gene deletion  Gene deletion  Gene deletion  Gene deletion  Gene deletion  Gene deletion  Gene deletion  Gene deletion  Gene deletion  Gene deletion  Gene deletion  Gene deletion  Gene deletion  Gene deletion  Gene deletion  Gene deletion  Gene deletion  Gene deletion  Gene deletion  Gene deletion  Gene deletion  Gene deletion  Gene deletion  Gene deletion  Gene deletion  Gene deletion  Gene deletion  Gene deletion  Gene deletion  Gene deletion  Gene deletion  Gene deletion  Gene deletion  Gene deletion  Gene deletion  Gene deletion  Gene deletion  Gene deletion  ChIP-PCR  ChIP-PCR  ChIP-PCR  ChIP-PCR  ChIP-PCR  ChIP-PCR  ChIP-PCR  ChIP-PCR |
